# Utility of Ascitic Fluid Adenosine Deaminase Levels in the Diagnosis of Tuberculous Peritonitis in General Medical Practice

**DOI:** 10.1155/2020/5792937

**Published:** 2020-04-22

**Authors:** Ayako Kumabe, Shuji Hatakeyama, Naoki Kanda, Yu Yamamoto, Masami Matsumura

**Affiliations:** ^1^Division of General Internal Medicine, Jichi Medical University Hospital, Yakushiji, Shimotsuke-shi, Tochigi 329-0498, Japan; ^2^Division of Community Medicine and Career Development, Kobe University Graduate School of Medicine, Arata-cho, Hyogo-ku, Kobe, Hyogo 652-0032, Japan; ^3^Division of Infectious Diseases, Jichi Medical University Hospital, Yakushiji, Shimotsuke-shi, Tochigi 329-0498, Japan

## Abstract

**Background:**

Tuberculous peritonitis is difficult to diagnose due to its varying clinical features, in addition to the low yield on bacterial culture or polymerase chain reaction using ascitic fluid samples. This study aimed to investigate the sensitivity and specificity of elevated adenosine deaminase (ADA) levels as a diagnostic marker for tuberculous peritonitis.

**Methods:**

A retrospective cohort of 181 adult patients who underwent ascitic fluid ADA level examination at Jichi Medical University Hospital between January 2006 and December 2015 were included. We collected data regarding ascitic fluid analyses including ADA levels, bacteriology and cytology, final diagnosis (cause of ascites), basis of the diagnosis, duration to diagnosis, and disease outcome.

**Results:**

Among 181 patients, elevated ascitic ADA levels (≥40 IU/L) were observed in 15 patients (median, 87.2 IU/L; range, 44.0–176.1 IU/L); 8 patients had tuberculous peritonitis, 4 had lymphoma-related ascites, and 2,had peritoneal carcinomatosis with bacterial coinfection, and 1 had chlamydial pelvic inflammatory disease. Among 166 patients without ascitic ADA level elevation (median, 7.3 IU/L; range, <2.0–39.1 IU/L), none had tuberculosis, 4 had lymphoma-related ascites, 28 had cancer/mesothelioma-related ascites, and 134 had ascites due to other causes. In our cohort, elevated ascitic fluid ADA levels (≥40 IU/L) showed 100% sensitivity, 96.0% specificity, 53.3% positive predictive value (PPV), and 100% negative predictive value for the diagnosis of peritoneal tuberculosis.

**Conclusions:**

Ascitic fluid ADA levels ≥40 IU/L showed excellent sensitivity, despite a low PPV, for the diagnosis of tuberculous peritonitis. Lymphoma-related ascites is an important mimic of tuberculous peritonitis that can result in high ascitic fluid ADA levels with similar clinical manifestations.

## 1. Introduction

The World Health Organization estimates that 10.0 million new cases of tuberculosis, equivalent to 133 cases per 100,000 population, occurred worldwide in 2017 [1]. The prevalence of tuberculosis varies across regions; Japan had 16,789 new tuberculosis cases (13.3 cases per 100,000 population) in 2017, the highest incidence among developed countries [2]. Tuberculous peritonitis accounts for less than 1% of all cases of tuberculosis [3]. Although rare, we should always pay attention to this disease, especially in endemic areas, because tuberculous peritonitis is difficult to diagnose, and diagnostic delays frequently occur.

Previous studies have demonstrated the usefulness of adenosine deaminase (ADA) measurements in the diagnosis of tuberculosis in body fluids, including cerebrospinal, pleural, peritoneal, and pericardial fluids [4, 5]. ADA levels in pleural effusion have been reported to increase in various causes of pleuritis, including tuberculosis, malignancy (carcinomas, leukemias, and lymphomas), rheumatoid arthritis, and systemic lupus erythematosus [5]. However, much fewer studies have evaluated the diagnostic value of ADA levels in ascitic fluid for tuberculous peritonitis [6, 7]. This study aimed to retrospectively assess the diagnostic usefulness of ascitic ADA tests for tuberculous peritonitis and differentiation from other causes of peritonitis.

## 2. Patients and Methods

We performed a retrospective observational study, including all adult patients who underwent ascitic ADA level examination at Jichi Medical University Hospital, Japan, between January 2006 and December 2015. This institute is a tertiary care hospital with 1,132 beds. Pediatric patients were excluded from the study. We reviewed the medical records of participants and collected data regarding ascitic fluid examination, final diagnosis (cause of ascites), basis of the diagnoses, presence, or absence of liver cirrhosis, time to diagnosis after symptom onset/admission, and disease outcome. The extracted data of ascitic fluid examination included ADA levels, cell count and differentials, serum-to-ascites albumin gradient, cytology, acid-fast bacilli (AFB) smear and culture, bacterial culture, and polymerase chain reaction assay for *Mycobacterium tuberculosis* (Tb PCR). We used the highest ADA level as the variable in this study if multiple measurements of ADA level were performed in a single patient.

The cut-off level of ascitic ADA in the diagnosis of tuberculous peritonitis was set at 40 IU/L according to previous studies [7, 8]. The diagnosis of peritoneal tuberculosis was made through a combination of pathological, microbiological, and/or clinical findings. This study was approved by the Ethics Committee of the Jichi Medical University (Number A17-038). The requirement for informed consent was waived owing to the retrospective design.

## 3. Results and Discussion

### 3.1. Results

**Figure 1:**
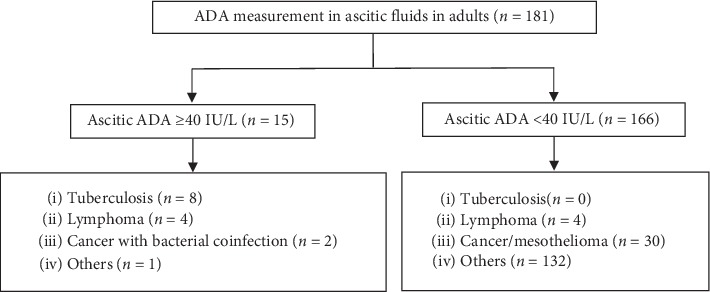
Diagnoses of ascites among patients who underwent ascitic adenosine deaminase (ADA) testing.

During the study period, 181 adult patients underwent ascitic fluid ADA level examination (Figure 1).

The median age was 62 (range, 18–85) years. One hundred patients (55.2%) were male. Most patients (98.9%) were Japanese, and the foreign patients were from China (*n* = 1) and Thailand (*n* = 1). Elevated ascitic ADA levels, which were defined as ≥40 IU/L, were observed in 15 patients (median, 87.2 IU/L; range, 44.0–176.1 IU/L). Among the 15 patients with high ascitic ADA levels, 8 (53.3%) were diagnosed with tuberculous peritonitis, 4 (26.7%) with lymphoma-related ascites, 2 (13.3%) with peritoneal carcinomatosis complicated with bacterial infection, and 1 (6.7%) with a chlamydial pelvic inflammatory disease (Figure 1 and Table 1). Among 166 patients without ascitic ADA level elevation, none had tuberculosis, 4 had lymphoma-related ascites, and 162 had ascites due to other causes including cancer/mesothelioma (*n* = 28), liver cirrhosis (*n* = 45), hypoalbuminemia (*n* = 16), and systemic lupus erythematosus (*n* = 12) (Figure 1). The median ascitic ADA level in the low-ascitic ADA cohort was 7.3 IU/L (range, <2.0–39.1 IU/L). Therefore, in this study, elevated ascitic fluid ADA levels (≥40 IU/L) showed 100% sensitivity, 96.0% specificity, 53.3% positive predictive value (PPV), and 100% negative predictive value (NPV) for the diagnosis of peritoneal tuberculosis.

The diagnoses of tuberculous peritonitis were established on the basis of bacteriologic and/or histopathologic results (7 patients) or clinically (1 patient). The positivity rates of culture, AFB smear, and Tb PCR tests in ascitic fluid samples were 25% (2 out of 8), 0% (0 out of 8), and 0% (0 out of 6), respectively. Among patients diagnosed with peritoneal tuberculosis, 6 patients (75.0%) were cured or experienced improved symptoms using anti-tuberculous agents, 1 (12.5%) died, and 1 (12.5%) was transferred to another hospital and was lost to follow-up.

Among the cases of lymphoma with high ascitic ADA levels (*n* = 4), 2 had cases of natural killer/T-cell lymphoma, 1 had a case of anaplastic large cell lymphoma (a rare type of T-cell lymphoma), and 1 had a case of B-cell lymphoma (Table 1). Among the patients diagnosed with lymphoma without ascitic ADA level elevation (*n* = 4), 2 had cases of B-cell lymphoma, 1 had a case of follicular lymphoma (a type of low-grade lymphoma developed from B-cells), and 1 had a case of peripheral T-cell lymphoma (Table 2). Although various types of lymphoma were observed in both cohorts, the mortality rate was higher among cohorts with ascitic ADA level elevation than among those without elevation (100% vs. 50.0%), and T-cell lymphoma rather than B cell lymphoma was predominantly observed in the cohort with elevated ascitic ADA levels (Tables 1 and 2).

Among 40 patients diagnosed with malignancy (carcinoma, lymphoma, and mesothelioma), elevated ascitic ADA levels were observed in 6 patients (15.0%); the majority (*n* = 4) had lymphoma and the rest (*n* = 2) had peritoneal carcinomatosis (one due to ovarian cancer and the other due to unknown primary cancer) with bacterial coinfection (Figure 1). Ascitic ADA level elevation was observed in 50.0% and 25.0% of patients diagnosed with lymphoma and ovarian cancer, respectively (Table 3).

### 3.2. Discussion

Tuberculous peritonitis has been difficult to diagnose due to its varying clinical features, in addition to the low yield on bacterial culture or PCR using ascitic fluid samples as observed in this study. Common symptoms of tuberculous peritonitis include abdominal pain, fever, and weight loss, which are not specific for tuberculosis. The sensitivity of AFB smear and culture using ascitic fluid samples for the diagnosis of tuberculous peritonitis has been reported as 0–6% and at most 20%, respectively [9–11]. Furthermore, mycobacterial culture is not appropriate for a prompt diagnosis because it typically requires more than 3–4 weeks of incubation. Surgical peritoneal biopsy might yield high culture positivity and pathology for a definitive diagnosis. However, this surgical procedure is sometimes too invasive to perform in the clinical setting.

ADA, an enzyme required for purine degradation, is distributed in systemic tissues and body fluids. ADA levels in the ascitic fluid have been reported as a potential marker for the diagnosis of tuberculous peritonitis, with a sensitivity of 100% and specificity of 97% in previous studies [6–8], and sensitivity of 100%, specificity of 96.0%, PPV of 53.3%, and NPV of 100% in this study. It is important for clinicians to understand the reason for this low PPV. The most important activity of ADA involves the proliferation and differentiation of T lymphocytes, and T lymphocytes have approximately 10 times higher ADA levels than B lymphocytes [4, 6, 7]. Therefore, ADA levels can theoretically increase in cases of effusion (including pleural, peritoneal, and cerebrospinal fluid) due to infection (especially tuberculosis), lymphoproliferative disorders, and rheumatic diseases. In our series, significant elevation of ascitic fluid ADA levels was strikingly observed in 2 diseases: tuberculous peritonitis and lymphoma. Although large studies are lacking, there have been anecdotal reports of ascitic ADA level elevation in patients with non-Hodgkin lymphoma [12, 13], ovarian cancer [14], and bacterial peritonitis [15].

Lymphoma was the second commonest disease associated with ADA level elevation in ascitic fluid in our cohort. Multiple cytology tests were useful for detecting lymphoma cells in the ascitic fluid. In this study, no malignancies except for lymphoma and ovarian cancer showed ADA level elevation in the ascitic fluid.

This study had several limitations. First, it was a retrospective, single-center cohort study that included a relatively small number of adult patients. Detection bias or ascertainment bias might be present, because physicians tend to test ascitic ADA levels more extensively when they consider tuberculosis as a differential diagnosis. Second, a single patient was included who was clinically diagnosed with peritoneal tuberculosis (in the absence of histological/bacteriological evidence). Therefore, the specificity of ascitic ADA level elevation for peritoneal tuberculosis might be overestimated.

## 4. Conclusions

Ascitic fluid ADA levels ≥40 IU/L showed an excellent sensitivity, despite a relatively low specificity, for the diagnosis of tuberculous peritonitis. Clinicians should be aware that both tuberculosis and lymphoma could result in high ascitic fluid ADA levels with similar clinical manifestations. Since yield on AFB smear, culture, or PCR testing using ascitic fluid samples is low, peritoneal biopsy should be considered for prompt and definite diagnosis of peritoneal tuberculosis.

## Figures and Tables

**Table 1:** Characteristics, causes, and consequences of patients with high ascitic ADA levels (≥40 IU/L).

	Ascitic fluid examination							Diagnosis		Outcome
	AFB smear	AFB culture	Tb PCR	Cell count (differential)	SAAG < 1.1	Cytology (class)	ADA (IU/L)					
Tuberculosis										
1	(−)	(−)	(−)	1,090/*μ*L (N 24%, L 69%)	No	II	44.0	Peritonitis	Histopathology of the peritoneal specimen	Lost to follow-up
2	(−)	(−)	(−)	130/*μ*L (L 99%)	Yes	II	76.1	Peritonitis, pleuritis	Culture for Tb of the pleural effusion	Improved
3	(−)	(+)	(−)	700/*μ*L (N 1%, L 96%)	Yes	I	87.4	Peritonitis	Culture for Tb of ascitic fluid	Cured
4	(−)	(−)	(−)	740/*μ*L (N 2%, L 90%)	Yes	U	102.2	Disseminated	Histopathology of the testis and culture and PCR for Tb of the urine	Cured
5	(−)	(−)	NT	1,640/*μ*L (N 12%, L 56%)	Yes	I	138.2	Peritonitis, pleuritis	Peritoneal nodules and PCR for Tb of the sputum	Improved
6	(−)	(−)	NT	46,400/*μ*L (N 96%, L 3%)	NT	I	176.1	Peritonitis	Clinical course	Cured
7	(−)	(−)	(−)	290/*μ*L (N 51%, L 40%)	NT	NT	57.3	Peritonitis, pulmonary Tb	Culture and PCR for Tb of the peritoneal specimen and sputum	Died
8	(−)	(+)	(−)	190/*μ*L (N 10%, L 83%)	NT	I	64.9	Peritonitis, pleuritis	Culture and PCR for Tb of the pleural effusion, urine, and sputum	Improved

Lymphoma										
9	NT	NT	NT	7,000/*μ*L (N 3%, A 97%)	NT	V	50.0	NK/T-cell lymphoma	Cytology of the ascitic fluid	Died
10	(−)	(−)	(−)	12,400/*μ*L (N 8%, L 9%, A 83%)	Yes	V	71.6	B-cell lymphoma	Cytology of the ascitic fluid	Died
11	(−)	(−)	(−)	570/*μ*L (N 29%, L 22%, A 41%)	Yes	V	157.9	NK/T-cell lymphoma	Cytology of the ascitic fluid and histopathology of the skin and lymph node specimens	Died
12	NT	NT	NT	8,500/*μ*L (N 14%, L 9%, A 73%)	NT	V	45.5	T-cell lymphoma	Cytology of the ascitic fluid and histopathology of the lymph node specimens	Died

Others										
13	NT	NT	NT	5,790/*μ*L (N 79%, L 2%, A 1%)	NT	IV	97.2	CP, BP	Cytology and bacterial culture of the ascitic fluid and histopathology of the liver specimen	Died
14	NT	NT	NT	84,250/*μ*L (N 81%, L 9%)	NT	V	153.0	CP, BP	Cytology and bacterial culture of the ascitic fluid	Died
15	(−)	(−)	(−)	112,000/*μ*L (N 54%, L 22%)	NT	I	87.2	PID	Nucleic acid amplification test for *Chlamydia trachomatis* of the endocervical specimen	Cured

AFB, acid-fast bacilli; Tb, tuberculosis; PCR, polymerase chain reaction; SAAG, serum-to-ascites albumin gradient; ADA, adenosine deaminase; NT, not tested; N, neutrophils; L, lymphocytes; A, atypical cell; U, unclassifiable; NK, natural killer; CP, carcinomatous peritonitis; BP, bacterial peritonitis; PID, pelvic inflammatory disease.

**Table 2:** Characteristics, causes, and consequences of patients with lymphoma without ascitic ADA level elevation (<40 IU/L).

	Ascitic fluid examination							Diagnosis		Outcome
	AFB smear	AFB culture	Tb PCR	Cell count (differential)	SAAG < 1.1	Cytology (class)	ADA (IU/L)					
Lymphoma without ascitic ADA level elevation (ADA <40 IU/L)										
16	NT	NT	NT	1,650/*μ*L (N 1%, L 95%)	NT	II	8.0	Follicular lymphoma	Cytology of the ascitic fluid and histopathology of the lymph node and bone marrow specimens	Remission
17	(−)	(−)	NT	2,690/*μ*L (N 12%, L 70%)	NT	III	15.4	Peripheral T-cell lymphoma	Histopathology of the lymph node and bone marrow specimens	Died
18	NT	NT	NT	7,960/*μ*L (N 8%, L 42%, A 35%)	NT	V	20.1	B-cell lymphoma	Cytology of the ascitic fluid	Remission
19	NT	NT	NT	1,150/*μ*L (N 10%, L 25%, A 51%)	Yes	V	17.2	B-cell lymphoma	Cytology of the ascitic fluid and histopathology of the lymph node specimens	Died

AFB, acid-fast bacilli; Tb, tuberculosis; PCR, polymerase chain reaction; SAAG, serum-to-ascites albumin gradient; ADA, adenosine deaminase; NT, not tested.

**Table 3:** Causative malignancy of ascitic fluid, by ascitic ADA level elevation.

Type of malignancy	Number of cases	
	Ascitic ADA ≥ 40 IU/L	Ascitic ADA < 40 IU/L
Lymphoma	4	4
Ovarian cancer	1	3
Gastric cancer	0	6
Pancreatic cancer	0	4
Peritoneal cancer	0	4
Lung cancer	0	3
Cholangiocarcinoma	0	2
Breast cancer	0	1
Endometrial cancer	0	1
Fallopian tube cancer	0	1
Hepatocellular carcinoma	0	1
Peritoneal mesothelioma	0	1
Retroperitoneal tumor	0	1
Cancer of unknown origin	1	2
Total	6	34

## Data Availability

The data used to support the findings of this study are available from the corresponding author upon request.
